# Risk factors for problem behavior in adolescents of parents with a chronic medical condition

**DOI:** 10.1007/s00787-012-0279-4

**Published:** 2012-04-28

**Authors:** Dominik Sebastian Sieh, Johanna Maria Augusta Visser-Meily, Frans Jeroen Oort, Anne Marie Meijer

**Affiliations:** 1 Research Institute of Child Development and Education, University of Amsterdam, Nieuwe Prinsengracht 130, 1018 VZ Amsterdam, Netherlands; 2 Rudolf Magnus Institute of Neuroscience, University Medical Center Utrecht, Heidelberglaan 100, 3584 CX Utrecht, Netherlands

**Keywords:** Adolescent, Chronic medical condition, Parent, Problem behavior

## Abstract

A wide array of risk factors for problem behavior in adolescents with chronically ill parents emerges from the literature. This study aims to identify those factors with the highest impact on internalizing problem behavior (anxious, depressed and withdrawn behavior, and somatic complaints) and externalizing problem behavior (aggressive and rule-breaking behavior) as measured by the Youth Self-Report (YSR). The YSR was filled in by 160 adolescents (mean age = 15.1 years) from 100 families (102 chronically ill parents and 83 healthy spouses). Linear mixed model analyses were used, enabling separation of variance attributable to individual factors and variance attributable to family membership (i.e., family cluster effect). Predictors were child, parent, illness-related and family characteristics. The results showed that almost half of the variance in internalizing problem scores was explained by family membership, while externalizing problems were mainly explained by individual factors. Roughly 60 % of the variance in internalizing problems was predicted by illness duration, adolescents’ feeling of isolation, daily hassles affecting personal life and alienation from the mother. Approximately a third of the variance in externalizing problems was predicted by adolescents’ male gender, daily hassles concerning ill parents and alienation from both parents. In conclusion, the variance in adolescent problem behavior is largely accounted for by family membership, children’s daily hassles and parent–child attachment. To prevent marginalization of adolescents with a chronically ill parent, it is important to be alert for signs of problem behavior and foster the peer and family support system.

## Introduction

The worldwide prevalence of chronic medical condition (CMC) affecting parents ranges between 4 and 12 % depending on the definition of illness and sample characteristics [1]. Focusing on the Netherlands, chronic parental illness affects approximately half a million children [2]. Advances in medical care and the growing number of older parents contribute to the increasing prevalence of children affected by parental CMC [3, 4]. In several European countries, the recent austerity measures have led to decreased financial support for people with CMC [5]. These measures augment the pressure on family members to provide caregiving, resulting in a higher prevalence of young caregivers.

Parental CMC has a tremendous impact on children [6]. Sieh et al. [1] conducted a meta-analysis on a total of 1,858 children, showing that growing up with a chronically ill parent poses an increased risk for problem behavior measured with the Youth Self-Report (YSR). This proves to be especially true for internalizing behavior (anxious, depressed and withdrawn behavior, and somatic complaints), but the overall effect size for externalizing behavior (aggressive and rule-breaking behavior) is significant as well. The YSR appears particularly sensitive to the types of problems faced by these vulnerable youth. Although protective factors and positive outcomes are relevant as well, this paper will focus on risk factors for internalizing and externalizing problems measured with the YSR because it is our priority to identify adolescents who need help. Internalizing problems refer to problematic behaviors that are directed toward the self, whereas externalizing problems denote projections of inner conflicts or unpleasant feelings toward external circumstances or other persons. In the normal population, girls are prone to elicit more internalizing behavior and less externalizing behavior than boys [7, 8]. In children with parental CMC, these gender differences seem to be less pronounced [1].

Categorically, research to date has demonstrated that increased problem behavior is related to child characteristics, parent characteristics, illness-related characteristics and family characteristics. Concerning child characteristics (gender, age, caregiving characteristics, caregiving impact and daily hassles affecting personal life), a meta-analysis found that effects for internalizing and externalizing problems were pronounced in studies including more girls and younger children [1]. Adolescents with parental CMC may have to take care of the household and other family members, restricting their leisure time, social life and school tasks, which may provoke problem behavior [9]. Meijer, van Oostveen and Stams [10] investigated 77 children of parents with Parkinson disease and ascertained that the frequency of caregiving tasks and negative feelings associated with caregiving significantly contributed to children’s problem behavior. Several empirical studies presented large effects for the relationship between adolescent problem behavior and variables capturing the impact of parental illness on children’s well-being. For example, a study on 100 children with a chronically ill or disabled parent concluded that variables measuring the caregiving impact (i.e., caregiving responsibilities, activity restrictions and feeling of isolation) were related to somatization, depression and anxiety [11, 12]. In a study on 81 children of parents with physical or mental illness, children’s feeling of isolation correlated with emotional symptoms and conduct problems [13]. Dufour, Meijer, van de Port and Visser-Meily [14] investigated children of parents with Parkinson disease or stroke and discovered that daily hassles affecting personal life were common and predicted stress in children.

Further, numerous studies on CMC have examined the relationship between children’s problem behavior and parent characteristics (gender, age, and depression of both parents, and caregiver strain of the healthy parent). Children’s internalizing and externalizing problems were more pronounced in studies with younger parents [1]. In addition, externalizing problems in adolescents were pronounced when the mother was ill and in studies marked by a high percentage of single parenthood. A less recent review on children with chronically ill or disabled parents concluded that children’s problem behavior was especially related to parental depression [9]. This may be understood considering that both parents of the target group are likely to suffer from increased depression scores compared to parents without CMC [15]. Further, healthy parents may experience caregiver strain that often involves being emotionally and/or physically less available, affecting adolescent outcomes. In a study on children of parents with stroke, caregiver strain of the healthy spouse emerged as a risk factor for children’s problem behavior [15].

A tradition in the field of parental CMC is to investigate whether children’s problem behavior is associated with illness-related characteristics (illness duration, ill parent’s health-related quality of life and unpredictability of parental illness). Sieh et al. [1] unraveled that studies characterized by long illness duration were positively related to both internalizing and externalizing problem behavior in children. Ireland and Pakenham [13] discovered that gradual illness onset contributed to poor youth adjustment. Not illness severity indices, but the perceived stressfulness of CMC proved to be connected to children’s problem behavior [9, 16]. Characteristics associated with the ill parent’s health-related quality of life, such as functional impairment, were not directly linked to children’s problem behavior [6]. Little is known about the unpredictability of parental illness, although it supposedly has an adverse impact on children [11].

With regard to family characteristics (socio-economic status, marital functioning, quality of parent–child attachment and children’s daily hassles concerning ill and healthy parents), Sieh et al. [1] ascertained that children’s problem behavior was more common in studies characterized by low socio-economic status (SES). A study on parental stroke affirmed that increased problem behavior scores were linked to the quality of marital relationship [15]. Further, Ireland and Pakenham [13] concluded that parent attachment security did not predict children’s emotional and behavioral outcome scores. On the contrary, Evans, Keenan and Shipton [17] examined children of mothers with chronic pain, concluding that insecure attachment was more common than in the control group. Children’s perception of daily hassles concerning ill and healthy parents appeared to be connected to stress and problem behavior in children of parents with Parkinson disease [10, 14], but no distinct conclusions about this relationship can be drawn thus far.

**Fig. 1:**
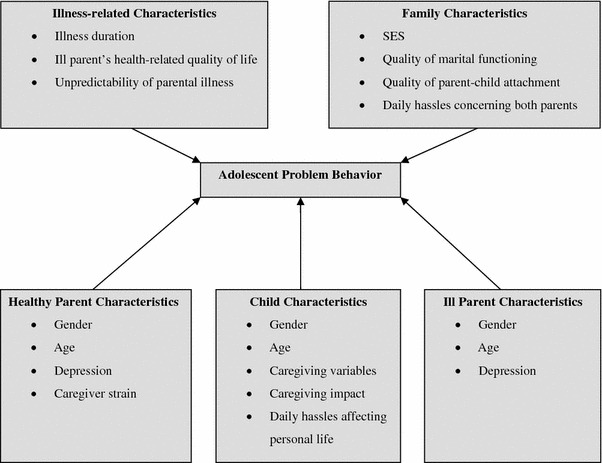
Theoretical model predicting adolescent problem behavior

Based on the body of the current literature, we have developed a predictive model of adolescent problem behavior (see Fig. 1). The theoretical origin of the model lies in the family systems theory [18–20], assuming that family members influence each other in multiple interactions and are interdependent. Because parental CMC has impact on family members for an extended period, it presumably elicits changes in family resources and adaptational processes of parents and children. As the family system is so dynamic, the number of factors and processes easily exceeds the number of cases, especially in research areas where large samples are difficult to recruit. In addition, processes like personal reactions to parental CMC are hard to measure in cross-sectional studies such as ours. We therefore choose an empirically driven model, assuming that internalizing and externalizing problem behavior is predicted by child characteristics, parent characteristics, illness-related characteristics and family characteristics [9].

Child characteristics are the core of the model. Next to child characteristics, the predictive model includes characteristics of ill parents and, if present, healthy spouses. Metaphorically speaking, illness-related and family characteristics overshadow adolescent outcomes. Illness-related characteristics not only include static characteristics like illness duration but also illness symptoms and functional impairment that are colligated with the ill parent’s quality of life. At last, family characteristics are an integral part of the model, including information about how the family functions as a whole, whether the family has financial and interpersonal buffers, and whether adolescents perceive daily hassles concerning their parents.

Research to date has frequently examined ill parents, spouses and children separately [1, 9]. Numerous studies overemphasize the importance of illness-related characteristics and merely examine a few risk factors for problem behavior. As a consequence, it remains unclear which factors constitute predictors for adolescent problem behavior when potential risk factors are examined simultaneously. In addition, most research has not taken into account that children in families share the same environment and may be similar to each other because of their family membership [21]. Consequently, possible dependencies between children from the same family (family cluster effect) should be considered.

Our first aim is to examine to which degree the variability in problem scores is accounted for at the individual level (variance between children) and at family level (variance between families), using multilevel modeling. In addition, we compare the target group to the Dutch normative sample of the YSR [8] to indicate effect sizes for internalizing and externalizing problems for girls and boys separately. Percentages of (sub)clinical cases of problem behavior in children with parental CMC compared to those of the normative sample are also presented. Second and most importantly, we aim to detect risk factors with the highest predictive value for problem behavior in the target group. As such, our study provides the basis for the development of a screening instrument for problem behavior.

Based on the literature [1, 9], we assume that demographic and illness-related characteristics have less impact on adolescent problem behavior than child, parent and family characteristics. Regarding child characteristics, we hypothesize that female gender of the child, young age of children, large caregiving impact and high frequencies of caregiving tasks and daily hassles affecting personal life are significant predictors of problem behavior [1, 9–14]. With respect to parent characteristics, we expect that high levels of parental depression and caregiver strain of the spouse are associated with adolescent problem behavior [1, 9, 15]. In terms of illness-related characteristics, we hypothesize that long illness duration and high unpredictability of illness are positively related to adolescent problem behavior [1, 6, 11, 13, 16]. Concerning family characteristics, we hypothesize that adolescent problem behavior is predicted by low SES, poor quality of parent attachment, low marital functioning and high frequency of daily hassles concerning both parents [1, 15, 17].

## Methods

### Procedure and participants

In this study, the whole family participated including children, parents with CMC and, if present, healthy spouses. Participants were recruited across the Netherlands in rehabilitation and community centers, hospitals, schools and public places (e.g., libraries) between September 2008 and April 2010. Besides, 30 randomly selected general health practitioners in all Dutch provinces were asked to cooperate and post brochures in their office. Finally, all major organizations for chronically ill patients in the Netherlands, such as the cardiovascular patient association, were asked to recruit potential participants. Once participants contacted the project manager, they received additional information about the purpose of the study design and participation. After initial screening of eligibility for participation over the phone, the participating families received an information package and informed consent form. After written informed consent had been given, a team of research assistants who were trained by the project manager made an appointment to administer questionnaires at the families’ homes. Adolescent participants received a cinema ticket worth 10 Euros. Participating families were informed about the project status on several occasions. The study was approved by the ethical commission of the Research Institute of Child Development and Education of the University of Amsterdam.

Only adolescents between 10 and 20 years of age who lived at home were included. Additionally, all participants had to speak sufficient Dutch to fill in the questionnaires worded in Dutch. Adolescents diagnosed with a severe chronic physical illness were excluded. One or both of their parents had to be diagnosed with a chronic medical condition lasting more than 6 months and causing functional impairment. A medical doctor in our team created a list of diagnoses that were unconditionally included (e.g., cerebral contusion). According to our criteria, CMC was associated with functional impairment of the ill parent that, however, was not assessed with a questionnaire prior to participation. Instead, we had a phone conversation with the chronically ill parent to inquire diagnostic information and the level of impairment.

Of the 116 families showing interest in participation, only 16 families were not part of the final sample, resulting in a high participation rate (86.2 %). Eight families dropped out without indicating a reason. One family indicated to perceive participation as a burden. The remaining families could not participate because their children were too old, too young or disabled, or the ill parent was not impaired. In two families, both parents were ill, leading to a sample of 100 families with 160 adolescents between 10 and 20 years of age, 102 ill parents and 83 healthy parents. Most families consisted of married parents or couples living together. Fifteen families were counted as single parent household, four of which were characterized by a long-distance relationship between the parents. Parental CMC included multiple sclerosis (28.4 %), rheumatoid arthritis (19.6 %), brain damage (16.7 %), neuromuscular disease (14.7 %), spinal cord injury (6.9 %), inflammatory bowel disease (5.9 %), Parkinson disease (4.9 %), and diabetes type I (2.9 %). Illness duration was longer than 10 years on average (see Table 1). More than two-thirds of the ill parents were female. While the majority of ill parents was unemployed, most spouses worked full-time. Almost all families (97 %) were native Dutch. Three families were originally from Germany, Hungary or Surinam.

**Table 1:** Demographics of children, parents and families

Children (*N* = 160)	
Female	51.9 %
Age in years (SD)	15.09 (2.34)
Living at home	100 %
Ill parents (*N* = 102)	
Female	67.6 %
Mean age in years (SD)	47.11 (5.50)
Illness duration in years (SD)	12.67 (11.05)
Mean number of all children per family (SD)	2.00 (1.02)
Currently employed	36.3 %
Healthy spouses (*N* = 83)	
Female	32.4 %
Mean age in years (*SD*)	47.46 (5.66)
Currently employed	84.3 %
Families (*N* = 100)	
Both parents diagnosed with CMC	2 %
Estimated net family income per month in Euros (SD)	2,685 (949)
Marital status/Living situation	
Married or living together	85 %
Mean duration of marital relationship in years (SD)	21.14 (4.68)

### Measures

#### Outcome measures

Internalizing and externalizing problem behavior in adolescents was measured with the YSR [22]. Items were summed to obtain a total score for internalizing symptoms (i.e., anxious/depressed behavior, withdrawn/depressed behavior and somatic complains) and externalizing symptoms (i.e., aggressive and rule-breaking behavior). In this study, Cronbach’s alpha showed excellent reliability for the internalizing scale (*α* = 0.91) and good reliability for the externalizing scale (*α* = 0.81). The YSR is mainly used for adolescents aged 11–18 but has also been administered to 10- and 19-year-old children in the validation study of the test makers [8]. Only 11 children of our sample were 10, 19 or 20 years of age. For this subsample, the reliability of internalizing problems was slightly higher (*α* = 0.93) than for the children aged 11–18 years (*n* = 149, *α* = 0.91). The reliability of externalizing problems was also better in children in the age of 10, 19 or 20 years (*α* = 0.86) than in the remaining 149 children (*α* = 0.81). Raw scores were transformed into standardized T-scores reflecting a mean population distribution of 50 and standard deviation of 10. T-scores were categorized into three levels: *clinical cases* (64 and above), *subclinical cases* (between 60 and 63), and *normal cases* (59 and below).

#### Child characteristics

Children’s demographic characteristics were gender and age. Children’s exact age was calculated by subtracting birth date by the current date. To measure caregiving characteristics comparable to those determined with the scales of the Young Caregiver of Parent Inventory (YCOPI) [11], we used the Dutch Caregiving Inventory (DCI), determining *frequency of household chores* (*α* = 0.66) and *frequency of caregiving tasks* (*α* = 0.75). Household chores referred to items like cleaning the house and putting out garbage, while caregiving involved activities like helping the ill parent get dressed, take medication and go to the toilet. Higher scores indicate higher frequency of household chores and caregiving tasks. The validity and reliability indices of the DCI proved to be satisfactory to good [10]. To capture the caregiving impact, adolescents filled in three scales from the Young Caregiver of Parent Inventory (YCOPI) which was originally designed and validated by Pakenham et al. [11] and (back)translated by native speakers for the Dutch version. We used the 8-item scales *caregiving responsibilities*, *activity restrictions*, and the 3-item scale *feeling of isolation*. High scores designated higher caregiving responsibilities, activity restrictions and more isolation, respectively. These scales showed good reliability in our sample (*α* = 0.76; *α* = 0.87 and *α* = 0.75, respectively). The Dutch Daily Hassles Questionnaire (DDHQ), a child-report measure, assessed *frequency of daily hassles affecting personal life.* Personal life was constituted by 8 items in the areas of social time with friends, school duties and job status (e.g., *How often does your home situation affect your homework?*). The DDHQ showed good validity and reliability coefficients in prior studies [10, 14] and good reliability in this study (*α* = 0.80).

#### Parent characteristics

Parents’ demographic characteristics were gender and exact age. Depressive symptoms in both parents were measured with the Beck Depression Inventory (BDI) [23]. Higher scores express more depressive symptoms. The reliability of this measure was *α* = 0.85 for ill parents and *α* = 0.86 for healthy parents. Spouses assessed their caregiving strain by means of the Caregiver Strain Index (CSI), a valid and reliable measure consisting of 13 items with higher scores indicating more strain [24, 25]. Cronbach’s alpha in this study was *α* = 0.84.

#### Illness-related characteristics

Illness duration was calculated as time difference between the date of investigation and the date of diagnosis. As an indication of health-related quality of life, ill parents filled in the Medical Outcome Study Short Form-36 including 6 health-related scales: *physical functioning* (10 items, *α* = 0.93), *social functioning* (2 items, *α* = 0.80), *role limitations due to physical health problems* (4 items, *α* = 0.79), *role limitations due to emotional problems* (3 items, *α* = 0.85), *bodily pain* (2 items, *α* = 0.86) and *general health perception* (5 items, *α* = 0.72). All scales range from 0 (*worst*) to 100 (*best*) [26]. Each participant of the family evaluated the *unpredictability of parental illness* by answering 5 items [11]. All items were summed and divided by the number of respondents per family, with higher scores indicating greater unpredictability. Cronbach’s alpha for the family scale with 15 items was *α* = 0.89.

#### Family characteristics

SES was evaluated as the monthly family income after tax deductions on an 8-point scale. The quality of parent attachment as reported by adolescents was determined with six 4-item subscales from the Inventory of Parent and Peer Attachment (IPPA) from Armsden and Greenberg [27]. Scales were *communication with mother* (*α* = 0.78), *confidence in mother* (*α* = 0.76), *alienation from mother* (*α* = 0.65), *communication with father* (*α* = 0.77), *confidence in father* (*α* = 0.83) and *alienation from father* (*α* = 0.77). Higher scores signal higher quality of communication, more confidence (mutual understanding, respect and confidence) and more alienation (estrangement, isolation and separation), respectively. The DDHQ was filled in by adolescents and was used to assess *frequency of* *daily hassles concerning ill parents* (6 items, *α* = 0.61) and *frequency of* *daily hassles concerning healthy parents* (5 items, *α* = 0.71). Both parents filled in 17 questions about the quality of marital relationship determined with the Interactional Problem Solving Inventory (IPSI) [28]. High scores represent high quality of marital relationship. Total scores were calculated as the sum of ill and healthy parents’ scores divided by 2. The reliability of the summed scale was *α* = 0.87.

### Statistical analyses

We calculated effect sizes [29] for problem behavior by subtracting the mean of the target group by the mean of the Dutch normative sample of the YSR [8] and dividing the outcome by the standard deviation of the normative sample. To explore, the relationships between adolescent problem behavior and all predictors were assessed with Pearson product moment and point-biserial correlations. Linear Mixed Modeling (LMM) was utilized to account for the family cluster effect, namely, that children within the same family are more similar to each other than children from different families, thereby violating the assumption of independence of observations. Through LMM, we can calculate the Intra Class Correlation (ICC) coefficient as a measure of the dependency of children within families [21].

In a specification search, we first included all predictors in LMM analyses. Subsequently, we removed predictors with non-significant effects stepwise, in reversed order of significance, until only significant effects remained (alpha = 5 %). Model fit was evaluated by calculating the Chi-square test as the difference between the log likelihood between two nested models. For completeness, we also report the Akaike Information Criterion [30] and Schwarz’s Bayesian Criterion [21]. At last, we calculated the explained variance of the predictor set in the final models. The assumptions for the statistical analyses were satisfied after checking for outliers and distribution. All analyses were conducted using SPSS, version 17.0.

## Results

### Raw and T-scores of problem behavior in adolescents

Only 5 % of the respondents had a few missing values which were substituted through Expectation Maximization, assuming missing at random [31]. Table 2 provides an overview of available descriptive statistics and the means and standard deviations of problem behavior in the target group and in the Dutch normative sample [8]. The normative sample consists of 11- to 18-year-old children who had not been referred to professional counseling until 12 months prior to assessment of the YSR. The distribution of gender in our sample and the normative sample were very similar. Our sample had a slightly higher SES than the normative sample, but this difference was small. For girls and boys in the target group, respectively, the effect sizes for internalizing problem behavior (Cohen’s *d* = 0.06; *d* = −0.06) were negligible or small [29]. However, compared to the Dutch normative sample, the percentages of clinical cases of internalizing behavior were elevated for both girls and boys. The effect for adolescents’ externalizing problem behavior was negative, meaning that girls and boys in the target group showed less externalizing problems than the normative sample (*d* = −0.48, *d* = −0.47, respectively).

**Table 2:** Comparisons between our sample of the target group and the Dutch normative sample on problem behavior

	Target group		Normative sample	
	83 girls (51.9 %)	77 boys (48.2 %)	521 girls (51.3 %)	495 boys (48.7 %)
Mean SES (SD)	4.91 (1.90)		4.50 (–)	
Age range	10–20 years		11–18 years	
Internalizing problems				
Raw score mean (SD)	11.06 (8.75)	8.01 (8.24)	10.64 (6.93)	8.35 (5.65)
T-score mean (SD)	49.93 (12.10)	48.31 (12.49)	50 (10)^a^	50 (10)^a^
Subclinical, *n* (%)	9 (10.8 %)	4 (5.2 %)	8.0 %	8.0 %
Clinical, *n* (%)	10 (12.0 %)	9 (11.7 %)	8.0 %	9.0 %
Externalizing problems				
Raw score mean (SD)	6.96 (5.27)	8.19 (5.45)	9.80 (5.90)	11.23 (6.41)
T-score mean (SD)	44.82 (10.01)	44.96 (9.53)	50 (10)^a^	50 (10)^a^
Subclinical, *n* (%)	2 (2.4 %)	2 (2.6 %)	8.0 %	8.0 %
Clinical, *n* (%)	3 (3.6 %)	4 (2.6 %)	8.0 %	9.0 %

SES *socioeconomic status*. In our study, SES was measured on an 8-point scale and in the Dutch normative sample, a 6-point scale was used according to Westerlaak, Kropman and Collaris [32]. We corrected the mean in the normative sample by a factor of 1.33 for the sake of comparability

^a^By Achenbach’s definition [22]

### Relationships between adolescent problem behavior and risk factors

#### Child characteristics

Girls and older adolescents displayed comparatively more internalizing problems (see Table 3). Children’s caregiving responsibilities and frequency of household chores were positively associated with internalizing problem behavior. High correlations with both problem behaviors were apparent for two of the variables measuring caregiving impact (i.e., activity restrictions and feeling of isolation) and for frequency of daily hassles affecting personal life.

**Table 3:** Correlations between predictors and adolescent problem behavior and explained variances of the predictors at the individual and family level

	Internalizing problem behavior			Externalizing problem behavior		
	Correlation	*R* ^2^ indiv. level	*R* ^2^ family level	Correlation	*R* ^2^ indiv. level	*R* ^2^ family level
Children						
Gender	0.18*	0.02	0.01	−0.11	0.01	0.02
Age	0.17*	0.02	0.03	0.10	0.00	0.02
YCOPI caregiving responsibilities	0.21**	0.04	0.05	0.08	0.13	0.12
YCOPI activity restrictions	0.49***	0.24	0.27	0.33***	0.04	0.04
YCOPI feeling of isolation	0.68***	0.45	0.48	0.35***	0.13	0.18
DCI frequency of household chores	0.22**	0.04	0.06	0.08	0.00	0.01
DCI frequency of caregiving tasks	0.07	−0.01	−0.01	0.08	0.00	0.00
DDHQ frequency of daily hassles affecting personal life	0.68***	0.45	0.49	0.41***	0.15	0.19
Parents						
Ill parent’s gender	0.02	−0.01	−0.01	0.06	0.00	0.00
Ill parent’s age	0.04	0.00	−0.01	0.01	0.00	0.01
Healthy parent’s age	0.07	0.00	0.00	0.03	−0.01	0.03
BDI (ill parent)	0.16*	0.02	0.02	0.14	0.01	0.02
BDI (healthy parent)	0.18*	0.03	0.07	0.10	0.01	0.04
CSI (healthy parent)	0.31	0.08	0.11	0.27	0.06	0.12
Illness						
Illness duration	0.06	0.00	−0.01	−0.06	0.00	−0.01
SF-36 physical functioning	−0.07	0.00	−0.01	−0.07	0.00	−0.01
SF-36 social functioning	−0.14	0.00	0.01	−0.13	0.00	0.02
SF-36 role limitations due to physical health	−0.02	−0.01	−0.01	0.01	0.00	−0.01
SF-36 role limitations due to emotional functioning	−0.17*	0.02	0.03	−0.12	0.00	0.01
SF-36 bodily pain	−0.21**	0.03	0.04	−0.14	0.01	0.02
SF-36 general health perception	0.09	0.00	−0.01	0.06	0.00	0.00
Unpredictability of ill parent’s condition	0.20*	0.03	0.05	0.11	0.00	0.00
Family						
SES	−0.12	0.01	0.02	−0.12	0.00	0.01
IPSI	−0.13	0.00	0.01	−0.18*	0.02	0.08
IPPA communication with mother	−0.24**	0.06	0.06	−0.30***	0.08	0.11
IPPA confidence in mother	−0.41***	0.16	0.19	−0.37***	0.13	0.18
IPPA alienation from mother	0.55***	0.29	0.33	0.49***	0.24	0.27
IPPA communication with father	−0.29***	0.08	0.09	−0.30***	0.08	0.11
IPPA confidence in father	−0.31***	0.09	0.10	−0.31***	0.09	0.12
IPPA alienation from father	0.38***	0.14	0.14	0.40***	0.16	0.18
DDHQ frequency of daily hassles (ill parent)	0.50***	0.24	0.24	0.36***	0.12	0.16
DDHQ frequency of daily hassles (healthy parent)	0.40***	0.16	0.19	0.23**	0.05	0.09

*Indiv.*  individual, *YCOPI* Young Caregiver of Parent Inventory, *DCI* Dutch caregiving inventory, *DDHQ* Dutch Daily Hassles Questionnaire, *BDI* Beck Depression Inventory, *CSI* Caregiver Strain Index, *SF-36* Medical Outcome Study Short Form-36, *SES* socio-economic status, *IPSI* Interpersonal Problem Solving Inventory, *IPPA* Inventory of Parent and Peer Attachment

Correlations are tested at a 5 % level of significance, without accounting for the inflation of familywise error rates. * *p* < 0.05; ** *p* < 0.01; *** *p* < 0.001. Significance tests are two-tailed

#### Parent characteristics

Depressive symptoms of ill and healthy parents were involved in a positive relationship with internalizing problem behavior. Spousal caregiver strain was positively related to both problem behaviors.

#### Illness-related characteristics

Internalizing problem behaviors correlated with parents’ role limitations due to emotional problems and bodily pain, and unpredictability of parental illness.

#### Family characteristics

The quality of marital relationship was negatively related to externalizing problem behavior. The quality of parent attachment and daily hassles concerning ill and healthy parents showed significant correlations with both problem behaviors.

### Explanatory models of problem behavior

In the empty model, the ICC for internalizing problem behavior was *ρ* = 0.44, meaning that 44 % of the total variance in internalizing problem scores was attributable to differences between families. For externalizing problem behavior, the ICC of the empty model was *ρ* = 0.19. For the predictor variables, explained variances at the individual and family level are listed in Table 3. Family characteristics generally explained more variance than other variables. The explained variances of adolescents’ feeling of isolation and frequency of daily hassles affecting personal life were also high. Overall, the sum of explained variances for internalizing problem scores was higher than for externalizing problem scores.

The models with multiple predictor variables (Table 4) are based on the data of 160 adolescents excluding variables reported by healthy spouses (age, depression, caregiver strain and marital functioning). For internalizing problem behavior, significant predictors were illness duration, adolescents’ frequency of daily hassles affecting personal life, feeling of isolation, and alienation from the mother. At the individual and family level, respectively, 58 and 61 % of the internalizing problem scores was explained by these predictors. The deviance test showed that the final model fitted the data better than the empty model [*χ* ^2^(4) = 127.74, *p* < 0.01]. Externalizing problem behavior was predicted by adolescents’ gender (male), daily hassles concerning ill parents and alienation from mothers and fathers. These predictors explained 34 and 42 % of the variability in externalizing problem scores at the individual and family level, respectively. The improvement in model fit was significant [*χ* ^2^(4) = 62.92, *p* < 0.01].

**Table 4:** Fixed and random effects of the predictors of internalizing and externalizing problem behavior in adolescents with a chronically ill parent

	Internalizing problem behavior				Externalizing problem behavior			
	Empty model		Final model		Empty model		Final model	
	Estimate	SE	Estimate	SE	Estimate	SE	Estimate	SE
*Fixed effects (effects assumed to be consistent across families)*								
Within family								
Child gender (male = 1, female = 2)							−1.88**	0.71
DHQ frequency of daily hassles affecting personal life			0.72***	0.15				
YCOPI feeling of isolation			1.03***	0.19				
DHQ frequency of daily hassles (ill parent)							0.26*	0.12
IPPA alienation from mother			0.94***	0.26			1.03***	0.20
IPPA alienation from father							0.53**	0.15
Between family								
Illness duration			0.11*	0.05				
Random effects (effects assumed to be variable across families)								
Intercept	9.70***	0.78	−2.79	1.52	7.51***	0.46	5.51	3.73
Within-family variance	41.34	7.14	20.45	3.46	23.75	4.02	19.22	3.13
Between-family variance	32.79	9.36	10.46	3.79	5.69	3.67	0.63	2.32
Explained variance (within family)			58.3 %				33.5 %	
Explained variance (between family)			61.3 %				41.7 %	
Fit indices								
Akaike Information Criterion	1,130.44		1,002.70		995.33		932.41	
Schwarz’s Bayesian Criterion	1,136.57		1,008.78		1,001.47		938.49	

*N* 160 adolescents, *N* 100 families, *YCOPI* Young Caregiver of Parent Inventory, *DCI* Dutch Caregiving Inventory, *DHQ* Daily Hassles Questionnaire, *CSI* Caregiver Strain Index, *SF-36* Medical Outcome Study Short Form-36, *IPPA* Inventory of Parent and Peer Attachment

* *p* < 0.05; ** *p* < 0.01; *** *p* < 0.001. Significance tests are two-tailed

To explore possible effects of healthy spouse variables, we excluded single parent families from the data set, fitting alternative models for 138 adolescents with two parents. For internalizing problem behavior, the extended model additionally contained adolescents’ frequency of caregiving tasks (regression estimate = −0.51, *p* = 0.01), caregiver strain of healthy parents (estimate = 0.41, *p* = 0.02), social functioning of ill parents (estimate = 0.04, *p* = 0.05) and adolescents’ daily hassles concerning ill parents (estimate = 0.56, *p* = 0.01). The extended model for externalizing problem behavior included the same variables as in the main model except for frequency of daily hassles concerning ill parents.

## Discussion

This study shows that the variance in adolescent internalizing problem scores was predicted by child characteristics (feeling of isolation and frequency of daily hassles affecting personal life), illness duration, and family characteristics (alienation from mother). These predictors explained the majority of the variability in internalizing problem scores of siblings within families and adolescents from different families. Concerning internalizing behavior, our findings supported the predictive model. Notably, a high extent of internalizing problem scores was explained by family membership. Thus, controlling for the family cluster effect was adequate and increased the accuracy of our predictive model. Parent characteristics did not act as direct effects on adolescent problem behavior, except for caregiver strain of the healthy spouse in the extended model. Externalizing problem behavior was predicted by child gender (male) and family characteristics (i.e., daily hassles concerning ill parents and alienation from parents). However, these predictors barely explained 35–42 % of the within-family and between-family variance in externalizing problem scores, meaning that the emerging risk factors did not predict adolescent problem behavior accurately. Our predictive model for externalizing problems seems to be less adequate because both parent and illness-related characteristics did not have direct effects. Additionally, the effects with significance explained a relatively low percentage of the variance in problem scores. In line with our hypotheses, demographic characteristics were less relevant than other characteristics with the exception of the finding that being a boy was a predictor for externalizing problems. This finding underlines the importance of examining possible gender differences regarding particular risk factors associated with externalizing behaviors.

The explained variances of certain child characteristics (i.e., feeling of isolation, activity restrictions and frequency of daily hassles affecting personal life) were high. These characteristics showed high correlations with both internalizing and externalizing problems. Concerning parent characteristics, the explained variances were low and only depression had a small positive link to internalizing problems. In regard of illness-related characteristics, the explained variances were also low and only a few characteristics had a significant correlation with children’s internalizing problems. Most family characteristics (e.g., quality of parent attachment and frequency of daily hassles concerning both parents) displayed high explained variances and strong associations with both problem behaviors. The fact that alienation from parents predicted both types of problem behavior suggests that parent attachment, specifically the degree of children’s estrangement, isolation and separation from the parent, is an important variable in the screening for adolescent problem behavior [3, 33, 34]. Our results provide more evidence for attachment theory than for our predictive model as depicted in Fig. 1.

According to attachment theory, parents play a significant role in comforting their children who feel threatened by stressful life events such as chronic illness. Especially when parents are too alienated to support their children, adolescents seem to develop problems [35]. It can be argued that attachment issues are a result of parentification, meaning that children feel and act like parents who care for other family members and become estranged from their father and mother due to the role reversal of caregiver and care receiver [36]. Attachment issues in our sample were reflected in high correlations between problem behavior and the quality of parent attachment (i.e., communication with parents, confidence in parents and alienation from parents). On the contrary, Ireland and Pakenham [13] found that attachment security towards the ill/disabled parent was not associated with child adjustment. They concluded that adolescent caregiving experiences and the attachment to the healthy spouse may moderate the effects of parent attachment. On several points, however, the study of Ireland and Pakenham differed from ours. Firstly, they also included parental mental illness and children were up to 25 years old. Secondly, although Ireland and Pakenham used the same instrument for parent attachment, they measured child adjustment with a much shorter questionnaire (i.e., Strengths and Difficulties Questionnaire) from Goodman, Meltzer and Bailey [37]. This instrument does not assess internalizing problems and may not be as sensitive as the YSR to detect specific adjustment problems in the target group.

In line with previous studies [9], the target group displayed a slightly increased risk for internalizing problems considering the percentages of clinical cases. To compare, in the Dutch normative sample of the YSR, only 8 % of girls and 9 % of boys are categorized as clinical cases, while these percentages are clearly higher in our sample (12 for girls and 11.7 % for boys). On the one hand, this could mean that the target group is better-off than what we expected. On the other hand, our findings may reach beyond the data. For example, it is possible that adolescents cope well in the sense that they do not appear to have developed internalizing problems but may experience other concerns. In addition, Ferdinand [38] demonstrated that the anxious/depressed scale of the YSR predicted DSM-IV disorders only moderately, so no psychiatric conclusions should be drawn about adolescents in our sample. It may be concluded that the relative predominance of internalizing problems incorporates reports of the target group being confronted with themes as loss, bereavement and unpredictability of parental health. This trend may also be a consequence of worries about caregiving responsibilities [12, 39]. The target group frequently recollects their childhood as growing up too fast, taking on responsibilities which interfere with leisure activities and involve fatigue, social isolation, vigilance and fears of having done something wrong [11, 40]. Concerning gender differences, girls scored in the subclinical spectrum of internalizing problems more frequently than boys. As such, our results are congruent with previous findings suggesting that especially girls experience anxieties about altered family roles and the recurrence of illness symptoms of their parent. Girls also fear becoming ill themselves [35]. Withal, we found no gender differences in clinical cases and problem scores. Regarding the finding that girls in the normal population show more internalizing behavior and less externalizing behavior than boys [7, 8], our results confirm that in children with parental CMC, gender differences are less pronounced [1]. Possibly, the impact of parental CMC evens up gender differences in the sense that both boys and girls are confronted with a life-event that may be life-threatening and highly stressful. Both girls and boys are frequently required to assist in caregiving tasks which are rather classified as an activity of girls. To support this notion, we exploratively tested gender differences in caregiving responsibilities, and frequency of household chores and caregiving tasks, and we found no significant effect.

In stark contrast, our sample displayed few externalizing problems, endorsing the idea that somatic complaints and anxious, depressive and withdrawn behavior constitute a specific problem area [1]. The fact that externalizing problems are rare suggests that adolescents with chronically ill parents are not a risk group by definition. It is plausible that our sample showed less externalizing problem because we included other medical conditions than studies presenting elevated levels on externalizing problems. For instance, Siegel et al. [41] may have found a significant effect for externalizing problems because they examined children within the last half year of a terminal diagnosis. Significant effect sizes are also observed in samples of children of parents with HIV [42, 43] and in non-cancer studies [1], so certain medical diagnoses of the parent may be linked to larger effects for externalizing problems in children. Another option is that internalizing problems buffer against developing externalizing problems. Adolescents with elevated levels of anxiety or fearfulness are possibly less prone to engage in risk behaviors that are included in the externalizing problem scale. Parental CMC is a life-event for the whole family. A possible explanation for the predominance of internalizing behavior in the target group is that children may learn about the limited life expectancy of their parent; they question issues like genetic heredity and may start worrying about their own fate. Internalizing behaviors presumptively correspond better to insecurities about personal integrity than externalizing behavior such as lashing out at others. In addition, it may be difficult to blame others because a chronic illness is not intentionally inflicted by another person and rather stresses children’s powerlessness. Another possible reason for the underrepresentation of externalizing problems is that children develop empathy being surrounded by a parent who can be weak and needy, and therefore, they see no justification to act out. Similarly, adolescents adopting caregiving responsibilities may perceive parental authority themselves and feel no need to break rules.

Our results have to be interpreted with some caution. First, when evaluating the significance of correlations in Table 3, we did not take possible inflation of the familywise error into account. However, if none of the correlations had actually been larger than zero, then the false discovery rate would have been two. Yet, for internalizing problems, 19 out of 32 correlations (59 %) were significant. For externalizing problems, 12 correlations (38 %) were significant. As the number of significant correlations for both internalizing and externalizing problem behaviors exceeded the number of chance results by far, we do not consider the significant results as chance results. Regarding the high number of predictors, it is questionable whether the sample size was large enough to fit the models. With low sample sizes, linear models are prone to overfitting the data, threatening the model generalizability. The amount of explained variance as indicated by R-squares may consequently be inflated [21, 44]. Second, the recruitment method and specific sample characteristics possibly influenced our results or induced selection bias. Only highly impairing medical conditions were considered, so the results do not apply to all adolescents with parental CMC. In addition, our sample was recruited through care institutions that provide access to health care. Most families had an adequate income and the average socio-economic status may be considered medium to high. Likewise, we asked the whole family to participate. It may be assumed that participating families were more cohesive because they were open enough to discuss illness-related matters. Children who participated may have benefited from the openness of parents to participate in a study that potentially uncovers sensitive issues. In like manner, children filled in the questionnaires with other family members in the same room or building. Although the research assistants clearly communicated the confidentiality of self-report, children may have felt inhibited because of the mere presence of their parents. We therefore believe that our sample may have lower problem scores than an average child with parental CMC. Further, our sample consisted of Caucasian families of Western culture. Chronic illness has a different meaning depending on how the culture identifies symptoms and decides what is to be considered deviant and adaptive behavior. Culture dictates both illness manifestation and resources allocated to the definition of illness [45]. Third, we did not classify illness into distinct types, for example into diagnoses with non-fatal, possibly fatal and fatal outcomes [46]. Within our diagnostic sample, illness type greatly varied because we included several different diagnoses. With such a mixed illness sample, it was difficult to generate large subsamples defined by illness type, and it was beyond the scope of our paper to test an illness classification system. Nevertheless, the influence of parental illness type on adolescent problem behavior could be of interest for future studies. Fourth, our study did not focus on positive outcomes, such as prosocial behavior, although some studies suggest that offspring encounter few problems [47, 48] and may benefit from their situation by developing caregiving skills that nurture their self-esteem and sense of identity [49]. Fifth, we did not include hetero-reported problem behavior, which may have led to the issue of common-method variance, meaning that self-reported behavior correlates more strongly with other self-reported behavior than with hetero-reported behavior [50–52]. Notwithstanding, the risk of common-method variance often is less important than assumed [53]. Last but not least, we did not compare the target group to children with no history of parental illness, meaning that we are unable to verify whether the emerging risk factors are specific for problem behavior in the target group. Future research should examine a culturally diverse sample, apply an illness classification system and make use of a comparison group. There should also be deeper focus on positive outcomes of the target group, such as caregiver competence, empathy and prosocial behavior. In addition, assessing problem behavior should be based on reports of multiple informants.

In sum, our study confirms that adolescents with a chronically ill parent display slightly more internalizing problems than other children [1]. This seems to be mainly due to adolescents’ feeling of isolation, daily hassles affecting their social and school life and a lower quality of mother–child attachment. Externalizing problems were not common in our sample, affirming that adolescents with chronically ill parents do not form a risk group by definition. On the one hand, externalizing problems are rare and research sustains that the target group is empowered by the experience of caring and can be defined as strong, resilient, hopeful and skilled [54]. On the other hand, our results suggest that internalizing problems constitute an idiosyncratic problem area with specific risks for the target group. Future research should focus on risk factors for adolescent problem behavior at the individual and family level, aiming to prevent long-term problems. Preventive steps are necessary to guarantee that parental illness does not pose an increased risk for problem behavior in children. Throughout the chronic stage of illness, it is recommendable to use a family-centered approach, focusing on strengths and needs of all family members [6]. Families may benefit from sustenance and hope for caregivers. It is important to promote emotional and social support within the family and from peers and professionals to improve the developmental prospect of children with a chronically ill parent.
